# A serious esophageal–mediastinal fistula successfully treated by endoscopic debridement and continuous irrigation

**DOI:** 10.1055/a-2575-3622

**Published:** 2025-04-17

**Authors:** Rongjuan Zhu, Jiyu Zhang, Qingfen Zheng, Lixia Zhao, Dan Liu, Bingrong Liu

**Affiliations:** 1 191599 Department of Gastroenterology and Hepatology, The First Affiliated Hospital of Zhengzhou University, Zhengzhou, China

A 62-year-old man was admitted to our hospital with chest pain, progressive dysphagia, and low grade fever for 3 days after eating duck meat. Endoscopy revealed a bone embedded in his upper esophagus ( Fig. 1 **a** ). After removal of the bone, an esophageal perforation was identified ( Fig. 1 **b** ). The patient developed a high grade fever and worsening chest pain 5 days later, and computed tomography and a further endoscopy showed an esophageal–mediastinal fistula and a mediastinal abscess with pus extravasation ( Fig. 1 **c** ; Video 1 ). Esophagography confirmed the esophageal–mediastinal fistula was 8.0 × 5.0 cm ( Fig. 2 ).

**Fig. 1:**
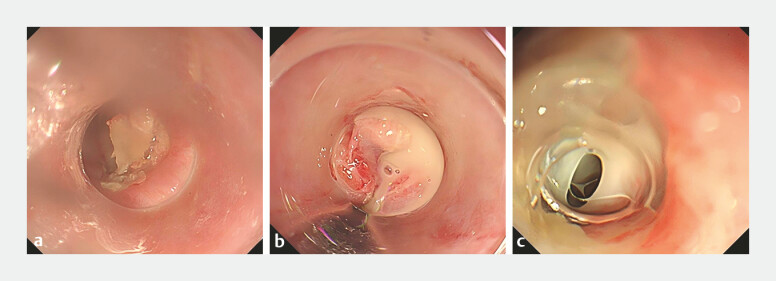
Endoscopic images showing: **a** a bone embedded in the upper esophagus; **b** an esophageal perforation filled with pus; **c** necrotic tissue and extravasation of pus in the fistula 5 days after removal of the bone.

A serious esophageal mediastinal fistula is treated by endoscopic debridement and continuous saline irrigation.Video 1

**Fig. 2:**
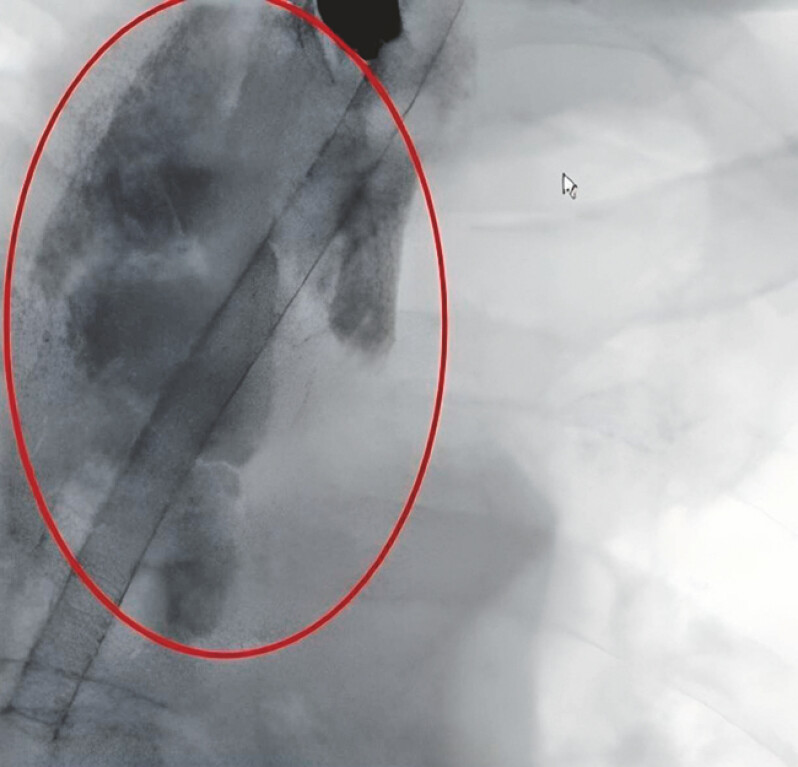
Esophagogram showing an esophageal–mediastinal fistula (8.0 × 5.0 cm).

To clean the fistula and control infection, we performed endoscopic debridement and inserted two tubes for continuous saline irrigation ( Video 1 ). A small caliber tube was used for continuous infusion of saline (1000 mL/24 hours), while the other large caliber tube was used for continuous suction. Additionally, a two-cavity tube was used to provide jejunal nutrition and esophageal decompression ( Fig. 3 **a** ). After 5 days, esophagography showed the fistula had decreased to 4.0 × 3.0 cm ( Fig. 3 **b** ). We removed two tubes, keeping the small caliber tube for continuous irrigation ( Fig. 3 **c** ), and allowed the patient to take an oral liquid diet. After 14 days, the final tube was removed and endoscopy confirmed that the fistula had healed ( Fig. 4 ). The patient remained healthy at follow-up after 16 months.

**Fig. 3:**
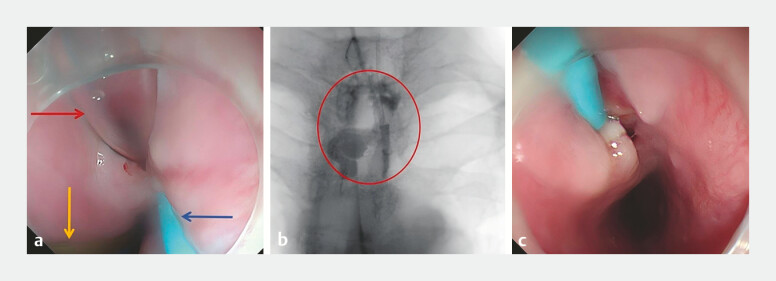
Images of the endoscopic debridement and continuous saline irrigation therapy showing: **a** two tubes inserted into the fistula cavity, a small caliber tube (blue arrow) for continuous saline infusion and a large caliber tube (red arrow) for continuous suction, plus a two-cavity tube (yellow arrow), with its distal end in the jejunum to provide nutrition and its proximal end in the esophageal lumen for decompression; **b** esophagogram after 5 days of therapy showing a reduction in the size of the fistula to 4.0 × 3.0 cm; **c** ongoing continuous saline irrigation via the small caliber tube only.

**Fig. 4:**
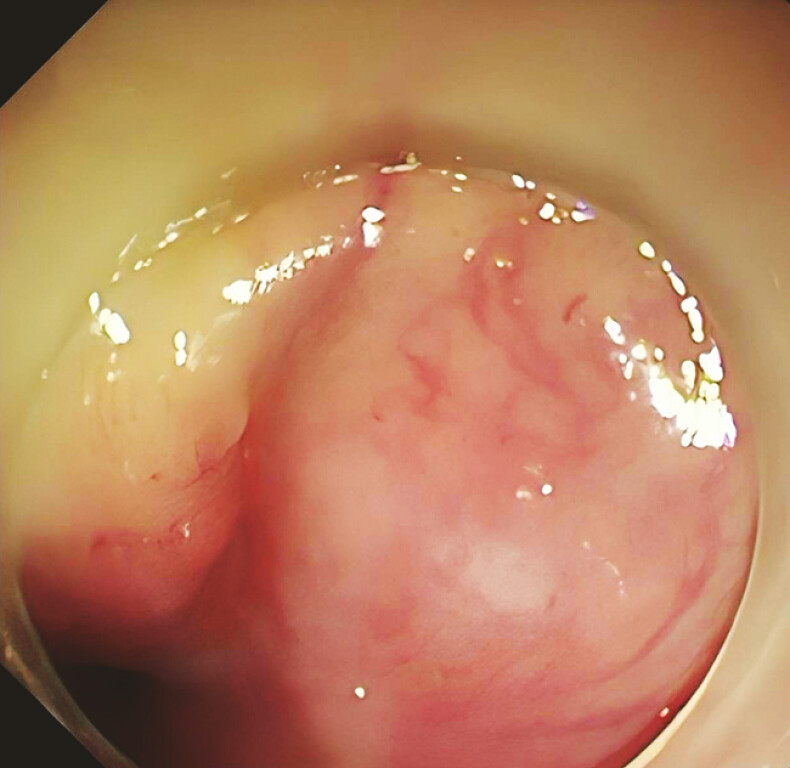
Endoscopic view after 14 days of therapy showing the healed fistula cavity.

Esophageal–mediastinal fistulas and abscesses have a high mortality rate 1 2 . Key treatments include pus drainage and infection control 3 . Endoscopic debridement effectively removes the necrotic tissue from the surface of the fistula, allowing the regeneration of fresh tissue and promoting healing. Continuous irrigation and suction can form a saline soak that facilitates the detachment and drainage of pus and necrotic tissue 4 . This case highlights this method as a rapid and effective therapy for esophageal–mediastinal fistulas and abscesses.

Endoscopy_UCTN_Code_TTT_1AO_2AI

## Notes

Endoscopy E-Videos https://eref.thieme.de/e-videos
*E-Videos* is an open access online section of the journal *Endoscopy* , reporting on interesting cases and new techniques in gastroenterological endoscopy. All papers include a high-quality video and are published with a Creative Commons CC-BY license. Endoscopy E-Videos qualify for HINARI discounts and waivers and eligibility is automatically checked during the submission process. We grant 100% waivers to articles whose corresponding authors are based in Group A countries and 50% waivers to those who are based in Group B countries as classified by Research4Life (see: https://www.research4life.org/access/eligibility/ ). This section has its own submission website at https://mc.manuscriptcentral.com/e-videos .
